# Development and Evaluation of a Novel-Thymol@Natural-Zeolite/Low-Density-Polyethylene Active Packaging Film: Applications for Pork Fillets Preservation

**DOI:** 10.3390/antiox12020523

**Published:** 2023-02-19

**Authors:** Constantinos E. Salmas, Aris E. Giannakas, Vassilios K. Karabagias, Dimitrios Moschovas, Ioannis K. Karabagias, Christina Gioti, Stavros Georgopoulos, Areti Leontiou, George Kehayias, Apostolos Avgeropoulos, Charalampos Proestos

**Affiliations:** 1 Department of Material Science and Engineering, University of Ioannina, 45110 Ioannina, Greece; 2 Department of Food Science and Technology, University of Patras, 30100 Agrinio, Greece; 3 Laboratory of Food Chemistry, Department of Chemistry, National and Kapodistrian University of Athens Zografou, 15771 Athens, Greece

**Keywords:** natural zeolite, thyme oil, thymol, low-density polyethylene, antioxidant film, pork fillets, food preservation, lipid oxidation, TBARS, heme iron

## Abstract

Sustainability, the circular economy, and the “greenhouse” effect have led the food packaging industry to use naturally available bio-compounds. The integration of such compounds in packaging films increases food safety and extends food shelf-life. The development of an active/antioxidant packaging film based on the widely commercially used low-density polyethylene, natural zeolite, and Thymol, a natural extract from thyme oil, is presented in this work. The obtained active films were characterized using X-Ray Diffraction, Fourier-Transform Infrared Spectroscopy, Scanning Electron Microscopy, and Differential Scanning Calorimetry techniques. The tensile strength, water–oxygen barrier properties, and total antioxidant activity were measured. Low-density polyethylene incorporated with Thymol@Natural Zeolite at a proportion of 15 wt% was the most promising material and was used as film to wrap-up pork fillets. The thiobarbituric acid (TBA) method and heme iron measurements indicated a delayed lipids oxidation using this film. A linear correlation between the TBA method and heme iron values seems to be established, which could result in a fast method to determine the degree of lipid oxidation in pork fillets. Finally, a two-stage diffusion process during Thymol release was observed, and the values of the diffusion coefficient was 2.09 × 10^−7^ and 1.21 × 10^−8^ cm^2^/s for each stage. The applied pseudo-second sorption model provided a rate constant k_2_ = 0.01647 (s^−1^). These results indicate the strong potential of such films to be used as food packaging materials free of E-number preservatives.

## 1. Introduction

The present need to enhance food safety and extend food shelf life promotes the development of new active food packaging materials [1,2]. Active packaging is defined as packaging that modifies the conditions of the packed food so as to extend its shelf life and improve its safety [3]. At the same time, the latest trend in food technology systems aims to reduce the chemical additives used and replace them with naturally abundant bioactive compounds such as essential oils (EOs), natural extracts, and their derivatives [4,5,6]. Furthermore, this trend in active packaging emphasizes the encapsulation of such natural bioactive components in packaging and their control release into food systems [4,7,8,9].

Low-density polyethylene (LDPE) is one of the most frequently used polymers for flexible packaging applications because of its excellent thermomechanical and barrier properties [10]. Although LDPE does not belong to the class of bio-based polymers and is not biodegradable, it could be replaced by bio-LDPE. The latter is considered biobased as it can be produced from renewable raw materials, specifically from bioethanol, which becomes ethylene after a dehydration process [11]. Among the natural antioxidant/antimicrobial agents that have been used, essential oils (EOs) and their components have been extensively investigated for application in active food packaging systems [9,12]. EOs exhibit significant antioxidant properties and have been investigated for their antibacterial properties against many food pathogens [4]. Although they are Generally Considered Safe (GRAS) as food additives, there are still many concerns about their direct addition to food. For this reason, the incorporation of EOs into food packaging films has been proposed, but their volatile nature results in their quick loss through evaporation. To overcome these drawbacks, it has been suggested to incorporate EOs on cheap and naturally abundant nanocarriers such as nanoclays to obtain bioactive EO–nanoclay nanohybrids. Then, such EO–nanoclay nanohybrids can be loaded onto polymer/biopolymer matrices to obtain active packaging films with a controlled release of EOs in food [13,14,15]. In this direction, montmorillonite clays and halloysite clays have been extensively investigated as nanocarriers for such biobased antioxidant/antimicrobial agents [16,17]. Natural zeolite (NZ) is a naturally abundant material suitable as an EO nanocarrier and has the advantage of being edible. Zeolites are made up of microporous crystalline aluminosilicates and they are excellent adsorbent materials. They can also be employed as antimicrobial materials and ethylene scavengers in food packaging [18,19,20,21]. Zeolites are known to be used as nanofillers to improve the gas barrier of LDPE [22]. Maleic anhydride grafted polyethylene (PE-g-MA) was used as compatibilizer to improve zeolite’s compatibility. The obtained LDPE/zeolite films achieved improved mechanical properties and lower water diffusivity and gas permeability in comparison to pure LDPE film. Moreover, the composite packaging film was able to maintain the quality and extend the shelf life of lime more than the LDPE packaging film [22]. LDPE–zeolite 4A films have also been prepared and used as CO_2_ adsorbents [23]. Antimicrobial films based on LDPE and 5 wt% zeolite modified with 1, 5, and 10 wt%. Ag ions have been recently developed [24]. In a previous report, a green preparation method was developed to adsorb a steam rich in thymol (TO) in NZ and thus obtain novel hybrid bioactive thymol–natural zeolite (TO@NZ) nanostructures [25]. Both NZ and the modified TO@NZ bioactive nanostructure were incorporated perfectly in a sodium alginate–glycerol polymer matrix and produced a very promising active film for food packaging with enhanced tensile and barrier properties, which could extend the shelf-life of soft cheese [25].

In this study, both pure NZ and modified TO@NZ nanohybrids at 5, 10, and 15 wt% nominal contents were loaded via an extrusion molding preparation method on LDPE. The obtained LDPE/xNZ and LDPE/xTO@NZ films (x is the wt% content of NZ or TO@NZ in the LDPE’s polymeric matrix) were characterized with X-Ray Diffraction (XRD) analysis, Fourier-Transformed Infrared (FTIR) spectroscopy, Scanning Electron Microscopy (SEM) images, and Differential Scanning Calorimetry (DSC) analysis. For all obtained LDPE/xNZ and LDPE/xTO@NZ films, tensile properties, water–oxygen barrier properties, and total antioxidant activity values were also determined using the 2,2-diphenyl-1-picrylhydrazyl (DPPH) assay method. The specific goal of the study is to demonstrate the optimal active LDPE/xTO@NZ film after characterizing all obtained LDPE/xNZ and LDPE/xTO@NZ films. TO diffusivity was calculated for the optimum LDPE/xTO@NZ film. Additionally, this optimum film was tested as an active packaging film to extend the shelf-life of “scallopini type” fresh pork meat fillets by estimating their lipid oxidation according to the thiobarbituric acid-reacting substances (TBARS) assay and heme iron content determination and comparing it to the lipid oxidation of fresh pork fillets packed with a LDPE/xNZ film and a pure LDPE film. Continuing from a previous work [26], the authors aim in this study to develop a food packaging film based on the most commercially used LDPE polymeric matrix, which will still be the basic packaging film for many years to come because of its properties, but with the addition of natural materials as antioxidants and E-number preservatives for food packaging.

## 2. Materials and Methods

### 2.1. Materials

Edible natural zeolite powder (≤20 μm, ≥650 m^2^/g, Serbian Micronized Zeolite, Product code HTSF257, Supplier Health Trade Athens, Greece) and thyme oil produced by Chemco (Via Achille Grandi, 13-13/A, 42030 Vezzano sul Crostolo RE, Italy) was purchased from a local market. LDPE was supplied by Sigma-Aldrich (CAS number of 9002-88-4, Sigma-Aldrich, St. Louis, MO, USA). Extra-pure DiPhenyl-1-PicrylHydrazyl (DPPH) was supplied by Sigma-Aldrich (Chemie GmbH Eschenstr. 5 82024 Taufkirchen, Germany) for analysis. Thiobarbituric acid (TBA) pro-analyze was purchased from Merck KGaA 64271 Darmstadt, Germany. Acetone 99% for analysis was supplied from Fisher Scientific, Bishop Meadow Road, Loughborough, LE11 SRG, UK. The local company Aifantis (Aifantis Group, Acheloos Bridge, Agrinio, Greece 30100) was the supplier of the “skalopini”-type pork meat. Three fresh samples of such meat without bones, weighing approximately 700 g each, were provided one hour after slaughter by the local company Aifantis.

### 2.2. Preparation of TO@NZ Hybrid Nanostructures

A novel distillation–adsorption process, which is reported elsewhere [25] and is based on the modification of a recent green adsorption method, was followed to produce a fraction of essential oils rich in thymol (TO) from thyme oil. Following this method, raw thyme oil was boiled in a spherical bottle to 180 °C for 15 min and the produced steam, rich in limonene and p-Cymene, was cooled down and liquified. Sequentially, the residue in the spherical bottle rich in thymol (TO) was boiled to 250 °C for 2 h, and the steam fraction produced, also rich in thymol (TO), was adsorbed and incorporated into the NZ [25].

### 2.3. Preparation of LDPE/xNZ and LDPE/xTO@NZ Films

An extrusion moulding process was followed for the preparation of LDPE/xNZ and LDPE/xTO@NZ films. A mini-lab twin extruder (Haake Mini Lab II, ThermoScientific, ANTISEL, S.A., Athens, Greece) was used for all experiments. The uniform extrusion temperature of the mini-lab twin extruder was set at 140 °C. The screw speed was set at 100 rpm, and the total processing time was set at 3 min. The total amount for the mini-lab twin extruder feed was set at 5 g of LDPE pellets and NZ or TO@NZ powder. To achieve 5, 10, and 15 wt% final nominal contents of NZ or TO@NZ on the obtained LDPE/xNZ and LDPE/xTO@NZ films, 4.75 g, 4.50 g, and 4.00 g of LDPE pellets was mixed with 0.25 g, 0.50 g, and 0.75 g of NZ or TO@NZ, respectively. The LDPE/xNZ and LDPE/xTO@NZ pellets obtained from the extrusion process were transformed into films by using a hydraulic press SPECAC GS15011 with heated platens (Specac Ltd. Limited. Registered Office: Science and Innovation Centre, Unit 12, Halo Business Park, Cray Ave, Orpington BR53FQ, registered in England No. 01008689). For each film, approx. 1.2 g of pellets of the LDPE/xNZ or LDPE/xTO@NZ batch were pressed at 110 °C and 2 MPa of constant pressure for 2 min.

### 2.4. Thyme Oil GC-MS Analysis

GC-MS analysis was carried out in a previous work [25] using a GC Ultra 2000/Finnigan Trace DSQ MSD instrument (Thermo Electron Corporation, Waltham, MA, USA) to analyze the as-received thyme oil as well as the remaining and collected fractions after the distillation process. Experimental conditions were also described in this report, and the identification of the compounds was performed by comparing the retention times and the mass spectra of volatiles to ADAMS, Wiley275, NIST, and in-house libraries [27,28].

### 2.5. Thermogravimetric Analysis (TG) Experiments of NZ and TO@NZ Nanohybrids

TG experiments were performed in NZ and TO@NZ hybrids using a Perkin-Elmer Pyris Diamond TGA/DTA instrument (Interlab, S.A., Athens, Greece) to estimate the total amount of TO adsorbed on NZ. Samples of approximately 5 mg were heated from 25 to 800 °C under N_2_ atmosphere at a heating rate of 5 °C/min.

### 2.6. Physicochemical Characterization of LDPE/xNZ and LDPE/xTO@NZ Films

Both the LDPE/xNZ and the LDPE/xTO@NZ films, as well as pure LDPE films, were physiochemically characterized using XRD analysis, FTIR spectroscopy, SEM images, and Differential Scanning Calorimetry measurements. XRD analysis was performed to investigate possible changes in crystallinity of LDPE/xNZ and LDPE/xTO@NZ films in comparison to pure LDPE film’s crystallinity. XRD measurements were performed in the range of 2° to 30° 2 theta by using a Brüker D8 Advance diffractometer (Brüker, Analytical Instruments, S.A., Athens, Greece) equipped with a LINXEYE XE high-resolution energy-dispersive detector. FTIR spectrometry measurements were carried out using an FT/IR-6000 JASCO Fourier-transform spectrometer (JASCO, Interlab, S.A., Athens, Greece) to investigate possible interactions between the NZ, TO@NZ, and LDPE matrix according to the methodology described previously [25]. SEM analysis as well as EDX analysis of films were performed to study the surface and cross-section morphology of both LDPE/xNZ and LDPE/xTO@NZ films using a JEOL JSM-6510 LV SEM Microscope (Ltd., Tokyo, Japan) equipped with an X-Act EDS detector from Oxford Instruments, Abingdon, Oxford shire, UK (an acceleration voltage of 20 kV was applied) according to the methodology described previously [25]. Finally, DSC measurements were carried out using a DSC214 Polyma Differential Scanning Calorimeter (NETZSCH manufacturer, Selb, Germany) in order to investigate possible changes in the T_g_ temperature of LDPE following the addition of both NZ and TO@NZ according to the methodology described previously [25].

### 2.7. Tensile Properties of Films

The tensile properties of plastic films were studied according to the ASTM D638 [29] method using a Simantzü AX-G 5kNt instrument (Simantzu. Asteriadis, S.A., Athens, Greece). In addition, the Young’s modulus, tensile strength (σ_uts_), and % elongation at break (%ε) values were calculated to investigate the effect of NZ and TO@NZ incorporation into the LDPE matrix. At least three to five “dog bone” samples (ASTM D638 type iv) of each film were tensioned at a crosshead speed of 2 mm/min while force (N) and displacement (mm) were recorded.

### 2.8. Water Vapor Transmission Rate Measurements and Water Diffusion Coefficient Calculation

The Water Vapor Transmission Rate (WVTR g·cm^−2^·s^−1^) for all LDPE/xNZ and LDPE/xTO@NZ films, as well as for pure LDPE film, was measured at 38 °C and 95 %RH using a handmade apparatus and employing the ASTM E96/E 96M-05 method. Circular film samples were placed on top of a one-open-end cylindrical tube made of plexiglass. The cylinder, which contained dried silica gel, was sealed with a rubber O-ring. The test tube was placed in a glass desiccator and stored in a Vevor XHC-25 chamber (Vevor company, China) under an environment of 95% relative humidity (RH) at 38 °C. Such conditions were obtained by placing 200 mL of saturated magnesium nitrate solution into the desiccator. Each tube was weighed periodically for 24 h. The WVTR values were calculated according to equations described in detail in a recent publication [25]. In this publication [25], the equation to obtain water vapor diffusivity (D_wv_) values was also described and used herein for similar calculations.

### 2.9. Oxygen Transmission Rate Measurements and Oxygen Permeability Calculation

Oxygen Transmission Rate (OTR) values (cc O_2_/m^2^/day) for all LDPE/xNZ and LDPE/xTO@NZ films, as well as for pure LDPE film, were measured with an oxygen permeation analyzer (O.P.A., 8001, Systech Illinois Instruments Co., Johnsburg, IL, USA) at 23 °C and 0% RH according to the ASTM D 3985 method. The oxygen permeability coefficient values (Pe_O2_) were calculated according to the equation provided and clearly described in [25].

### 2.10. Total Antioxidant Activity Assay of LDPE/xNZ and LDPE/xTO@NZ Films Surface

Total antioxidant activity assay was performed for all LDPE/xNZ and LDPE/xTO@NZ films, as well as for pure LDPE film, using the diphenyl-1-picrylhydrazyl (DPPH) method [30,31]. Briefly, 100 mg of each film was placed inside a dark bottle and 10 mL of 40 ppm DPPH ethanol solution was added. A sample of 10 mL of 40 ppm DPPH solution placed in a dark bottle with no film was used as reference. For all samples, the initial absorbance and the absorbance after 24 h of incubation were measured at 517 nm using a Jasco V-530 UV-vis spectrophotometer (Artisan Technology Group **^®^** 101 Mercury Drive Champaign, IL 61822, USA). Three samples of each film were measured to achieve the statistical mean as the final measurement. The % antioxidant activity after 24 h of incubation of the active films was calculated according to Equation (1):

(1) $$\%\;\mathrm{Antioxidant}\;\mathrm{activity}=\frac{{Abs}_{ref}-{Abs}_{sample}}{{Abs}_{ref}}\times 100$$

### 2.11. Calculation of TO Release Diffusion Coefficient: Pseudo-Second-Order Desorption Process

The accelarated migration of TO from active films was based on the method descibed by Darvish M. et al. [32], with some modifications. For the release experiments, a moisture analyzer AXIS AS-60 (AXIS Sp. z o.o. ul. Kartuska 375b, 80-125 Gdańsk) was employed. Circular samples of LDPE/15TO@NZ film with average diameters of 3 cm were placed inside the moisture balance chamber and a thermogravimetric program was applied by raising the temperature up to 70 °C. The film mass was recorded every 120 s. The temperature of 70 °C was chosen for two reasons: (a) it is a temperature lower than the temperature where LDPE softens (80 °C) and (b) the aim was to simulate the conditions during thermal processing of foods packaged with such LDPE/15TO@NZ active packaging film and calculate the TO release content. For comparison, the same thermogravimetric programm was run for circular samples of the LDPE/15NZ film which was used as blank film. The mass loss of LDPE/15NZ film during the thermogravimetric program was attributed to the release of the adsorbed water vapor, and these values were substracted to obtain the mass loss values of LDPE/15TO@NZ film to calculate the net mass loss of TO molecules released from the film. For the statistical reliability of the measuraments three samples of each film were measured. The diffusion coefficient (D_TO_) for TO release out of the developed films process, was calculated according to the following Equation (2):

(2) $$\frac{m_{t}}{m_{\infty }}=\sqrt{4\frac{D\times t}{\pi \times \ell^{2}}}$$

where m_t_ and m_∞_ are the amount of TO released form the film after time t and after equilibrium time t_eq_→∞, respectivelly, D is the diffusion coefficient, and *l* is the average film thickness.

The linearization of Equation (6) leads to the slightly modified Equation (3):

(3) $${\left(\frac{m_{t}}{m_{\infty }}\right)}^{2}=4\frac{D\times t}{\pi \times \ell^{2}}$$

Moreover, the desorption rate was estimated using the common pseudo-second-order sorption mechanism model, which is reported in [33] and described by the following equation:

(4) $$q_{t}=\frac{q_{e}^{2}\times k_{2}\times t}{q_{e}\times k_{2}\times t+1}$$

where q_t_ = m_t_/m_0_ and q_e_ = 1.

### 2.12. Packaging Preservation Test of “Scaloppini”-Type Fresh Pork Meat Fillets

Fresh pork fillets, 80–90 g each, were packaged aseptically between two LDPE films (control sample), LDPE@15NZ films, and LDPE@15TO@NZ active films. The diameter of such films was 11 cm, and they were folded in meat-packaging paper without the inner paper film. Samples were prepared for testing after 2, 4, 6, 8, 10, and 12 days of storage for all packaging systems tested. After packaging, the fillets were placed in a preservation chamber at 4 ± 1 °C and then measured for lipid oxidation and heme iron content.

### 2.13. Lipid Oxidation of Packaged “Skalopini”-Type Fresh Pork Fillets

#### 2.13.1. Thiobarbituric Acid Reactive Substances

Thiobarbituric acid-reactive Substances (TBARS) were determined using the Jasco V-530 UV-vis spectrophotometer (Artisan Technology Group **^®^** 101 Mercury Drive Champaign, IL 61822, USA) according to the procedure described in a recent paper [26] and following the concept reported in other previous publications [34,35]. TBARS measurements were based on the adsorption Abs_538_ at λ = 538 nm and were expressed as mg of malondialdehyde per 1 kg of sample (mg_MDA_/kg_sample_) according to the following equation:

TBARS (mg_MDA_/kg_sample_) = 7.8 × Abs_538_ (5)

where 7.8 is a calibration constant to transform the absorbance Abs_538_ to mg_MDA_/kg_sample_ [35].

#### 2.13.2. Heme Iron Content

The heme iron content was determined according to the method reported by Clark et al. [36], with slight modifications. More specifically, 4 g of pork fillets was homogenized with a mixer (Vicko S.A, Athens, Greece) and adding 18 mL of acidified acetone. Then, the solution was left to stand at 25 °C, protected from light, for 1 h. Afterwards, the solution was filtered, and the absorbance was measured at 640 nm using the Jasco V-530 UV-vis spectrophotometer (Artisan Technology Group **^®^** 101 Mercury Drive Champaign, IL 61822, USA). The amount of heme iron in the pork-fillets was calculated according to the following equation:

Heme iron (μg/g) = Abs_640_ × 680 × 0.0882 (6)

where Abs_640_ is the absorbance value measured at λ = 640 nm, 680 is a calibration constant to convert the Abs_640_ to ppm concentration (μg_hematin_/g_sample_) if the prepared solution was made following the method of [37,38], and 0.0882 is the constant value to transform μg_hematin_ to μg_HFe_.

### 2.14. Calculation of Melting-Crystallization Enthalpy and the % Crystallinity of LDPE

Integrating the melting and crystallization peaks of the DSC plots, we obtained the ΔH (J/g) enthalpy of the melting and crystallization processes of the mixed LDPE/xNZ or LDPE/xTO@NZ mass of films. The ΔH_LDPE_ (J/g) enthalpy of the melting or crystallization process of the net LDPE mass of films is calculated using the following equation:

(7) $${\Delta H}_{LDPE}\left(\frac{J}{g}\right)=\frac{\Delta H(\frac{J}{g})}{1-x}$$

where x is the mass fraction of NZ or TO@NZ nanostructure in the film.

The %X_C_ is the percentage of the crystalline part of the mass of LDPE to the total LDPE mass, which is calculated using the following equation:

(8) $$\%X_{C}=100\times \frac{{\Delta H}_{LDPE}(\frac{J}{g})}{{\Delta H}_{100}(\frac{J}{g})}$$

### 2.15. Statistical Analysis

All measurements considering structural and mechanical properties were carried out on at least three different samples. The final results, which are presented in tables in this work, are the mean values of such measurements. Standard deviation is also presented in these tables and “equal means” hypothesis tests were executed. For such statistical treatment, SPSS ver. 20 software was used (IBM SPSS Statistics, IBM Corp., 1 New Orchard Road Armonk, New York 10504-1722, USA) and the ANOVA method with the assumption of confidence intervals C.I. = 95% and significance level *p* = 0.05 was adopted. Furthermore, equality (EA%) or inequality (IA%) assurance of means was tested according to the empirical equations reported in previous works [32]. In all cases, the mean values of properties for different kinds of samples were found to be statistically different, with an assurance factor greater than 52%.

## 3. Results and Discussion

### 3.1. GC-MS Results

The results of the GC-MS analysis are shown in Tables S1–S3 of the Supplementary Materials. Table S1 shows the GC-MS analysis results of thyme oil as received. Table S2 shows the GC-MS analysis results of the collected after the distillation process of the thyme oil fraction rich in limonene and p-Cymene. Table S3 shows the GC-MS results of the fraction of thymol remaining after the distillation process of the thyme oil. The results (see Table S1) show that as-received thyme oil consists of 12.3% Cymene, 15.5% limonene and 56.7% TO. The thymol fraction collected after the distillation process of thyme oil (see Table S2) consists of 32.0% Cymene, 31.9% limonene, and 21.0% TO. The remaining thymol fraction after the distillation process (see Table S3) consists of 86.7% TO and almost all limonene and Cymene disappeared. So, the GC-MS results show that the distillation process followed successfully resulted in a thyme oil fraction rich in TO that was then used in the adsorption process for the modification of pure NZ to obtain TO@NZ nanohybrids.

### 3.2. Physicochemical Characterization of TO@NZ Nanohybrids

**Figure 1:**
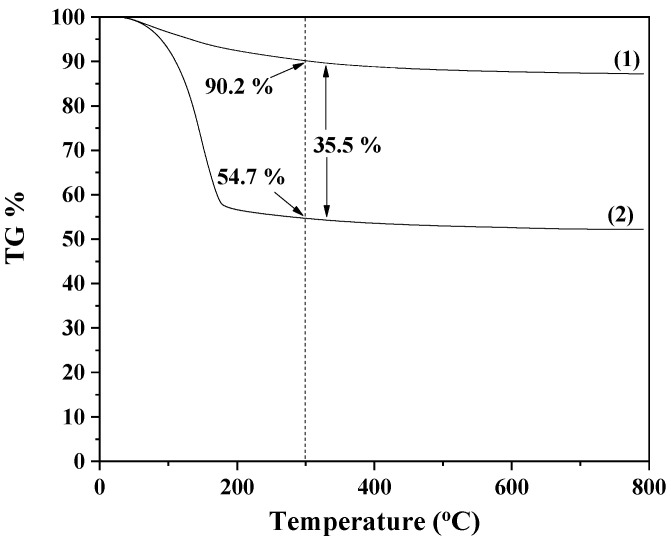
TG plots of pure NZ (1) and obtained TO@NZ (2) nanohybrids.

As we have recently shown [25] via Differential Scanning Calorimetry (DSC214 Polyma Differential Scanning Calorimeter, NETZSCH manufacturer, Selb, Germany) experiments, TO was the main fraction of molecules adsorbed on the NZ surface in the obtained TO@NZ nanohybrid. XRD analysis showed no differences in the crystallinity of the obtained TO@NZ nanohybrid as compared to pure NZ. The FTIR, spectrometry of the obtained TO@NZ nanohybrids revealed that the TO fraction is physiosorbed rather than chemisorbed on the surface of the NZ. The physisorption of such bioactive compounds on the NZ’s surface is favorable for the application of such nanohybrids in controlled release processes [25]. Moreover, in Figure 1, the TG plot of pure NZ as well as of the obtained TO@NZ nanohybrid are shown for comparison. For both NZ and TO@NZ, a mass loss step is obtained starting at about 100 °C and ending at about 500 °C. This mass loss is much higher for TO@NZ than pure NZ because of the desorption of both water and TO molecules. Considering that the boiling point of TO is 232 °C, we can assume that at 300 °C, the adsorbed TO will be totally desorbed. At this temperature, we calculate the mass loss of the TO@NZ nanohybrid and pure NZ (see Figure 1). By subtracting the mass loss of the TO@NZ nanohybrid at 300 °C from the mass loss of pure NZ at 300 °C, we calculated the amount of TO adsorption on NZ, which was found to be 35.5 wt%.

### 3.3. Physicochemical Characterization of the LDPE/xNZ and the LDPE/xTO@NZ Films

**Figure 2:**
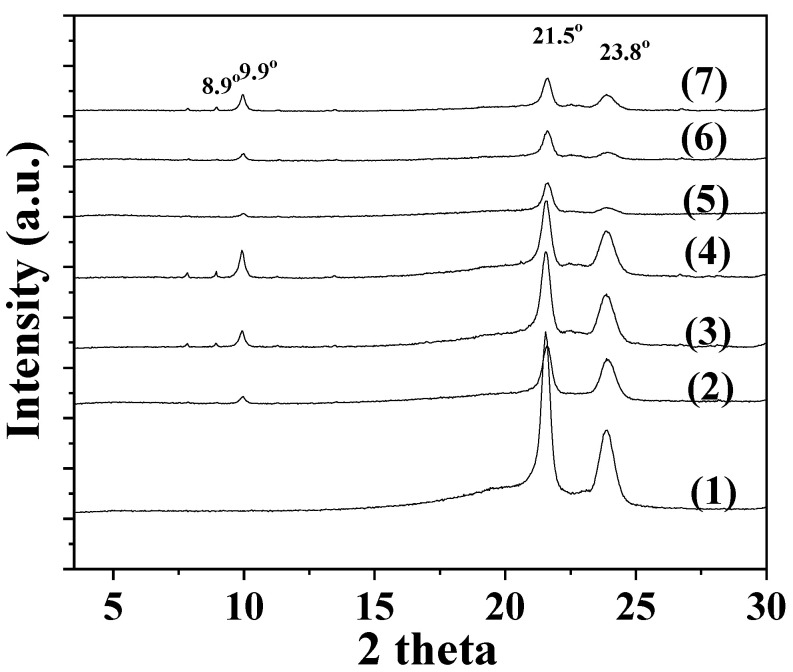
XRD plots of (1) pure LDPE, (2) LDPE/5NZ, (3) LDPE/10NZ, (4) LDPE/15NZ, (5) LDPE/5TO@NZ, (6) LDPE/10TO@NZ, and (7) LDPE/15TO@NZ films.

Figure 2 presents the XRD plots of all LDPE/xNZ (see plots (2), (3), and (4)) and LDPE/xxTO@NZ films (see plots (5), (6), and (7)), as well as pure LDPE films (see plot (1)) for comparison.

As it is observed, XRD plots of all films showed two well-defined peaks of LDPE at Bragg angles 2θ  =  21.5° and 23.8° [39]. In addition, the peaks at around 8.9° and 9.9° observed in the plots of all LDPE/xNZ and LDPE/xTO@NZ films are attributed to Heulandite Ca(Si_7_Al_2_)O_16_·6H_2_O monoclinic crystal phase (PDF-41-1357) of NZ [25]. In general, it is observed that LDPE’s peaks decreased in all LDPE/xNZ and even more so in LDPE/xTO@NZ films compared to the pure LDPE. Additionally, as the NZ and TO@NZ content increased the NZ’s peaks increased too. NZ’s peaks are less prominent in LDPE/xTO@NZ films compared to LDPE/xNZ films, which implies a higher dispersion of the TO@NZ nanostructure compared to the pure NZ in the LDPE matrix. No shift in LDPE’s peak is observed for either LDPE/xNZ or LDPE/xTO@NZ films. This implies that the blending of both NZ and TO@NZ with LDPE is physical and does not affect the structural morphology of the LDPE’s matrix.

**Figure 3:**
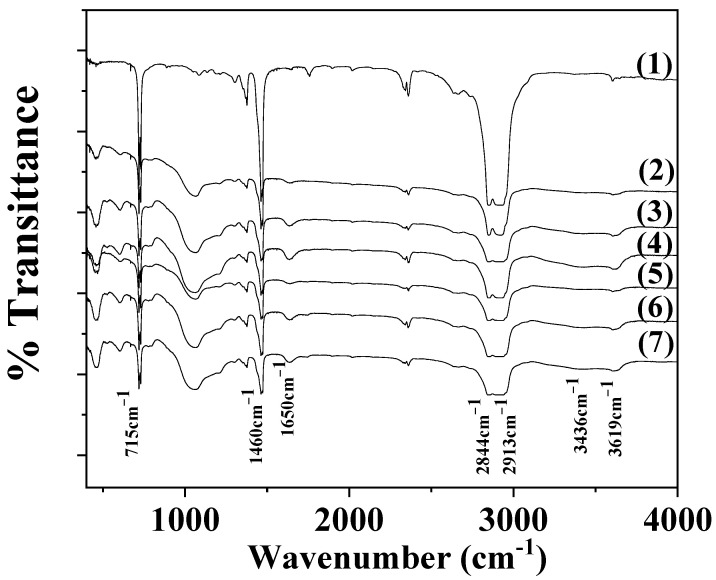
FTIR plots of (1) pure LDPE, (2) LDPE/5NZ, (3) LDPE/10NZ, (4) LDPE/15NZ, (5) LDPE/5TO@NZ, (6) LDPE/5TO@NZ, and (7) LDPE/5TO@NZ films.

Figure 3 shows the FTIR plots of pure LDPE and all LDPE/xNZ and LDPE/xTO@NZ films for comparison.

In the FTIR plots of both LDPE/xNZ and LDPE/xTO@NZ films, as well as for the pure LDPE films, the characteristic peaks of LDPE and NZ are mainly observed [13,25]. More specifically, at 1460 and 715 cm^−1^, asymmetric stretching of CH_3_ group, wagging of the CH_2_ group, and rocking of CH_2_ group are observed in LDPE. At 2913 and 2844 cm^−1^, symmetric stretching peaks of the CH_2_ group are observed in LDPE [13,40]. The bands at 3619 and 3436 cm^−1^ are assigned to the OH group stretching mode of NZ. The band at 1650 cm^−1^ corresponds to the OH group bending mode of the NZ, the band at 1090 cm^−1^ corresponds to the Si-O stretching vibration of NZ, and the band at 468 cm^−1^ corresponds to the -SiO_4_- bending mode of NZ. The absence of a shift in LDPE’s peaks when both NZ and TO@NZ nanostructures are added to the LDPE matrix means that no chemical bonding between the NZ and TO@NZ and LDPE chains is observed. Moreover, the absence of possible peaks in TO molecules in the FTIR plots of all LDPE/xTO@NZ films means that the TO molecules are blended in the inside surface of the LDPE/xTO@NZ films [25].

The surface/cross-section morphology of the pure polymer matrix LDPE and the hybrid nanocomposite films of LDPE/xNZ and LDPE/xTO@NZ were investigated using an SEM instrument equipped with an EDS detector, and the results confirmed that the NZ and hybrid nanostructure TO@NZ were homogeneously dispersed in the polymer matrix. EDS elemental analysis was carried out, and mapping from the surface of the pure and nanocomposite active packaging films was recorded in order to identify the chemical elements of the relative final materials.

**Figure 4:**
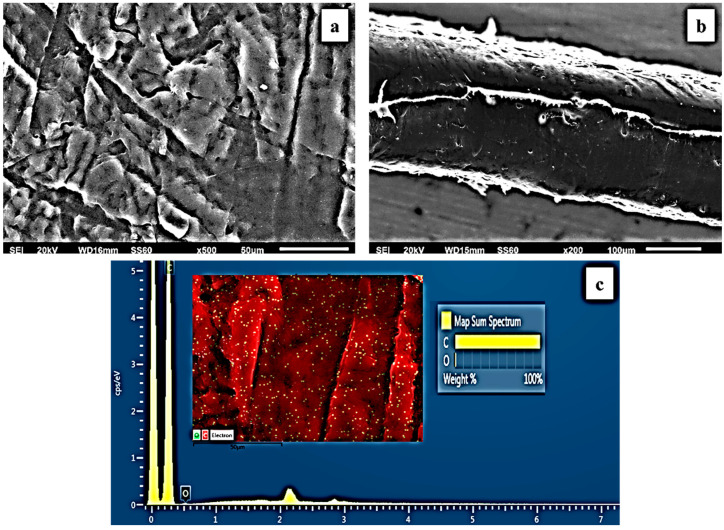
(**a**) SEM images of surface and (**b**) cross-section for the pure LDPE film. (**c**) Energy-dispersive spectrometer (EDS) spectra, relative elemental analysis, and mapping of the surface from the SEM image at ×1000 magnification.

The SEM images in Figure 4a,b exhibit the expected smooth and clear morphology of the pristine polymer. In Figure 4c, the EDS spectra certify the existence of carbon (C) and oxygen (O).

**Figure 5:**
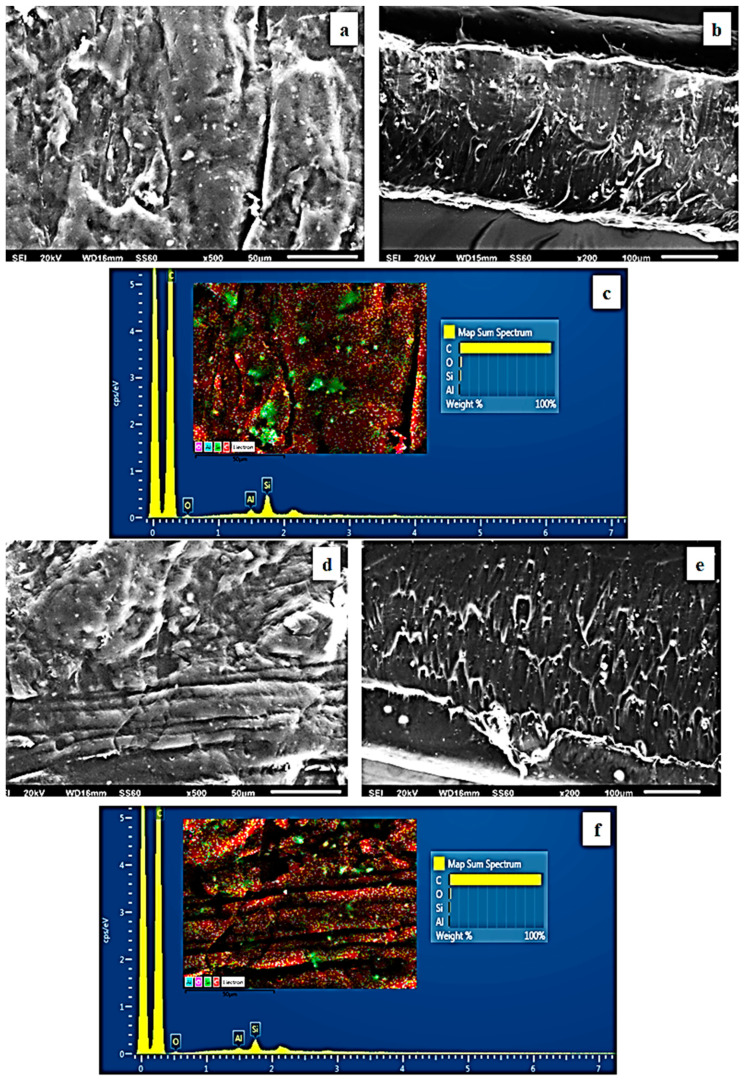
(**a,d**) SEM images of the surface and (**b**,**e**) cross-section of the nanocomposite films of LDPE/5NZ (**a**,**b**) and LDPE/5TO@NZ (**d**,**e**), respectively. (**c**,**f**) Energy-dispersive spectrometer (EDS) spectra, relative elemental analysis, and mapping of the surface from the SEM images at ×1000 magnification.

**Figure 6:**
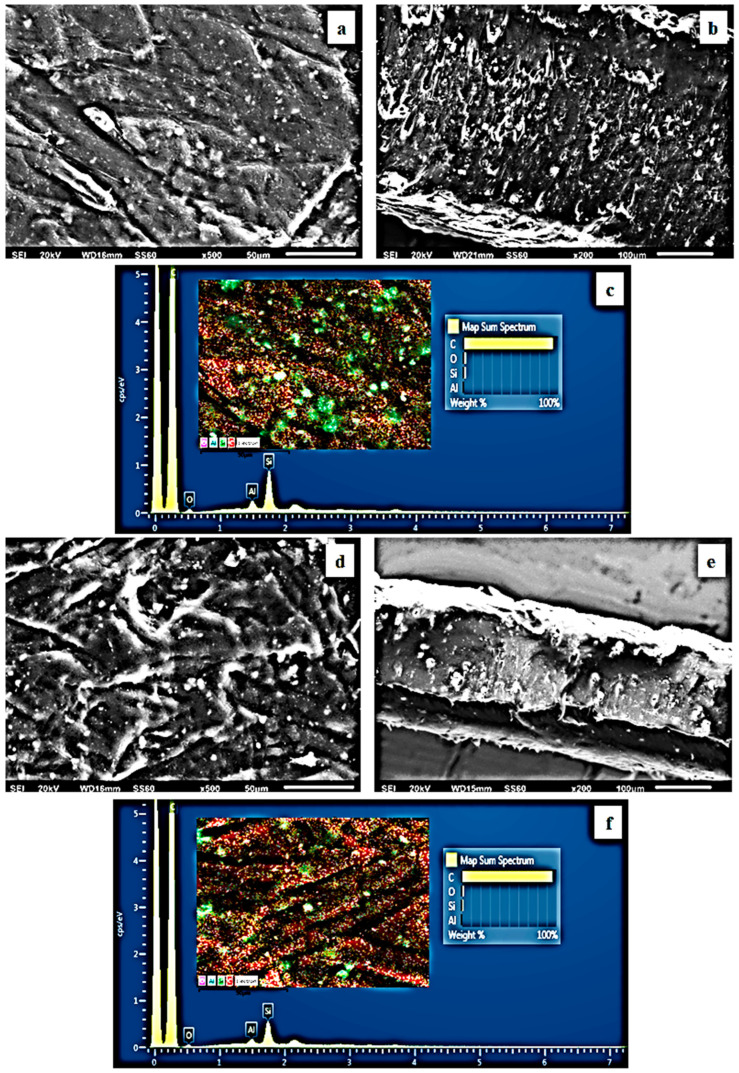
(**a**,**d**) SEM images of surface and (**b**,**e**) cross-section of the nanocomposite films of LDPE/10NZ (**a**,**b**) and LDPE/10TO@NZ (**d**,**e**), respectively. (**c**,**f**) Energy-dispersive spectrometer (EDS) spectra, relative elemental analysis, and mapping of the surface from the SEM images at ×1000 magnification.

**Figure 7:**
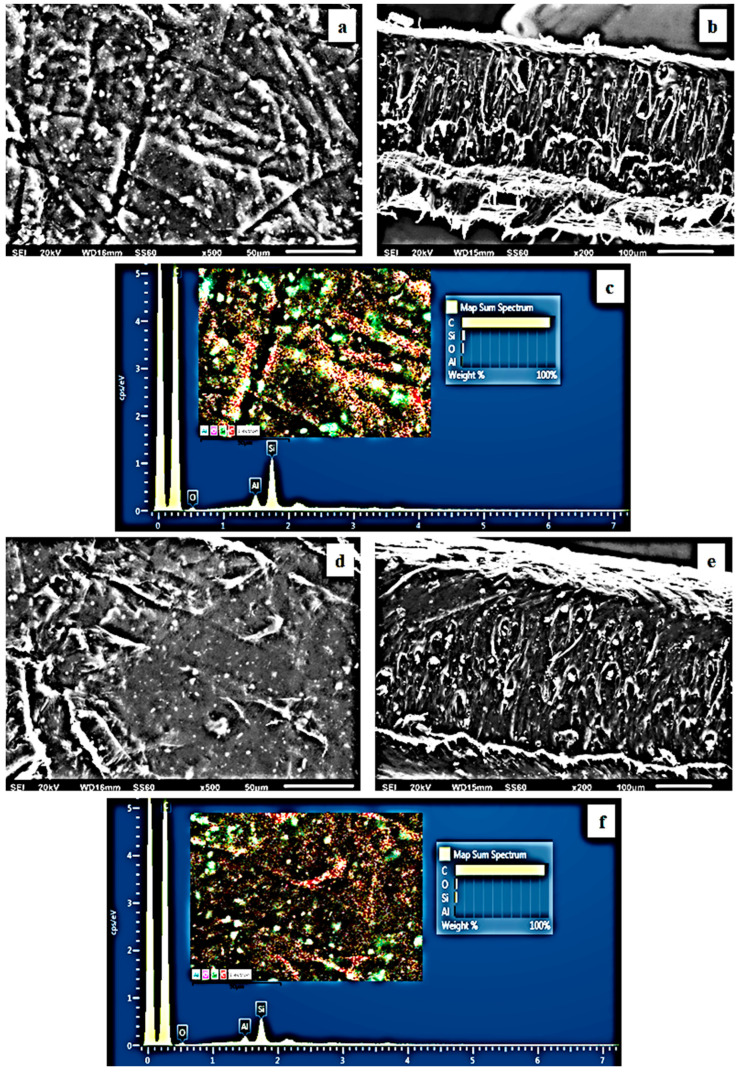
(**a**,**d**) SEM images of surface and (**b**,**e**) cross-section of the nanocomposite films of LDPE/15NZ (**a**,**b**) and LDPE/15TO@NZ (**d**,**e**), respectively. (**c**,**f**) Energy-dispersive spectrometer (EDS) spectra, relative elemental analysis, and mapping of the surface from the SEM images at ×1000 magnification.

In order to show the contrast with nanocomposite active films, the EDS spectra in Figure 5c,f, Figure 6c,f and Figure 7c,f show a chemical analysis that identified typical elements such as C, Si, Al, and O present in the packaging films with different concentrations (5, 10, 15%) of the TO@NZ hybrid nanostructure and pure NZ (white dots can be clearly identified in the surface of the LDPE matrix).

Images of the surface, relative cross-section, and chemical mapping of LDPE/xNZ and LDPE/xTO@NZ with different ratios of NZ and TO@NZ are presented in Figure 5, Figure 6 and Figure 7. It is obvious that the increase in content (after the incorporation of NZ and TO@NZ) in the nanocomposite materials increases the degree of aggregation accordingly. Nevertheless, the results of the SEM studies of the final nanocomposite films confirmed that the nanohybrids were homogeneously dispersed, which indicates their enhanced compatibility with the polymer matrix.

It should be mentioned, based on the SEM (surface and cross-section) studies and chemical mapping, that a significant difference is observed when the TO@NZ hybrid nanostructure is incorporated into the polymer matrix, as better interfacial adhesion and homogenous dispersion are evident compared to the corresponding nanocomposite film with pure NZ.

**Figure 8:**
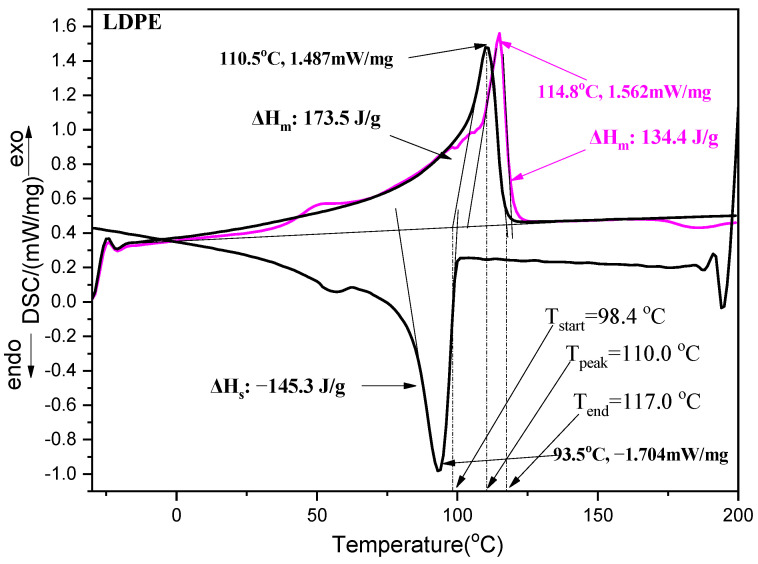
DSC plot of pure LDPE following a standard method saved in the library of the DSC214 Polyma instrument. The pink line is the first melting process (melting 1st step), the black downward line is the sequential crystallization process (crystallization 2nd step), and the black upward line is the last melting process (melting 3rd step).

Figure S1 presents the DSC plots of all LDPE/xNZ, LDPE/xTO@NZ films, as well as the DSC plot of the pure LDPE film. From these plots and according to the methodology described previously [41,42], some critical factors for the crystallization and melting processes of the films have been calculated and listed in Table 1. Scanning parameters, such as the rate of temperature increase and decrease, scanning temperature limits, number of iterations of the melting and crystallization processes, etc., were determined automatically by the chosen instrumental method (DSC214 Polyma NETZSCH manufacturer, Selb, Germany) for DSC measurements of LDPE materials. The DSC results are presented numerically in Table 1 and graphically in Figure 8. Such results are calculated using Equations (7) and (8) mentioned above, assuming that the ΔH_100_ (J/g) parameter for polyethylene is 293 (J/g) [43,44,45]. Moreover, T_start_ is the temperature at which the relevant peak starts to rise, T_peak_ is the maximum of each peak, and T_end_ is the temperature at which the relevant peak ends.

With a careful glance at the values listed in Table 1, it can be concluded that the addition of NZ and TO@NZ to LDPE (a) has no significant effect on the melting temperature of LDPE, (b) decreases the degree of crystallinity of LDPE in general, and (c) decreases the enthalpies of the crystallization and melting processes of films in general. Only the LDPE/10TO@NZ film exhibits crystallinity and enthalpy values equal to pure LDPE film. The decrease in the degree of crystallinity and both the crystallization and melting enthalpy values of LDPE with the addition of both NZ and TO@NZ are similar to previously described results concerning the addition of various contents of organically modified nanoclay to the LDPE matrix [46]. It should also be emphasized that DSC results for such LDPE/xNZ and LDPE/xTO@NZ nanocomposite films are reported here for the first time.

### 3.4. Tensile Properties of LDPE/xNZ and LDPE/xTO@NZ Films

Table 2 lists the calculated values of the modulus of elasticity €, ultimate strength (σ_uts_), and elongation at break %ε for all LDPE/xNZ and LDPE/xTO@NZ films, as well as for pure LDPE film for comparison.

As can be observed in Table 2 for all LDPE/xNZ and LDPE/xTO@NZ films, the modulus of elasticity values are increased in comparison to those of the pure LDPE film. Taking into account the standard deviation values of the ultimate strength values, it is concluded that the ultimate strength is unchanged rather than increased or decreased for all LDPE/xNZ and LDPE/xTO@NZ films in comparison to the ultimate strength of the pure LDPE film. Concerning the % elongation at break, it is observed that the %ε values are quite similar for LDPE/5NZ films and pure LDPE films, while for the LDPE/10NZ and LDPE/15NZ films a dramatic decrease in the obtained %ε values is observed in comparison to the %ε value of pure LDPE film. In contrast, for LDPE/5TO@NZ and LDPE/10TO@NZ films, the %ε values increase significantly up to 90.0% and 89.1%, respectively; for the LDPE/15TO@NZ film, the %ε value is 37.4% lower than the %ε values of LDPE/5TO@NZ and LDPE/10TO@NZ films but still higher than the %ε value of the pure LDPE film. These results on the mechanical properties of LDPE/xTO@NZ films indicate that the modification of NZ with TO enhances the dispersion and ability of TO@NZ nanohybrids in the LDPE matrix and that the TO molecules have a plasticization role in the obtained LDPE/TxO@NZ films. Jung et al. [23] also prepared a LDPE/zeolite 4A film via the twin extrusion process by varying the wt% of zeolite 4A content from 10 to 40% and studied their mechanical properties. With the increasing wt% of zeolite 4A content, a decrease in the ultimate strength values and a dramatic decrease in the % elongation at break values were recorded [23]. These behaviors are similar to the results presented here for LDPE/xNZ films. As mentioned in the introduction, no studies with films based on LDPE and NZ modified with EOs have been published in the literature. So, the mechanical properties presented here for LDPE/xTO@NZ films are being reported for the first time.

### 3.5. Water–Oxygen Barrier Properties of LDPE/xNZ and LDPE/xTO@NZ Films

In Table 3, the obtained water vapor transmission rate (WVTR) and oxygen transmission rate (OTR) values for all LDPE/xNZ and LDPE/xTO@NZ films, as well for pure LDPE films, are listed for comparison. Also in Table 3, the calculated water vapor diffusion coefficient (D_wv_) values and oxygen permeability (Pe_O2_) values are listed.

As observed in Table 3, the D_wv_ values are increased for all LDPE/xNZ films compared to the D_wv_ value of the pure LDPE film. This behavior is expected when an inorganic material such as NZ is dispersed inside a hydrophobic polymer matrix such as LDPE [40,47]. On the contrary, the D_wv_ values are decreased for all LDPE/xTO@NZ films as compared to pure LDPE film. This indicates that the modification of NZ with TO results in hydrophobic TO@NZ nanohybrids with a higher dispersion capability in the LDPE’s hydrophobic matrix This was also confirmed by the SEM image results shown above. The lowest D_wv_ value is for LDPE/5TO@NZ film and is equal to 1.14 *×* 10^−4^ cm^2^/s. This value is 62.7% lower than the D_wv_ value of the pure LDPE film, which is equal to 3.06 *×* 10^−4^ cm^2^/s.

On the other hand, Pe_O2_ values are significantly decreased for all LDPE/xNZ films, as well as for all LDPE/xTO@NZ films, as compared to pure LDPE films. Furthermore, LDPE/5TO@NZ, LDPE/10TO@NZ, and LDPE/15TO@NZ films obtained slightly lower Pe_O2_ values as compared to LDPE/5NZ, LDPE/10NZ, and LDPE/15NZ films, respectively. Thus, the LDPE/15TO@NZ film exhibits the lowest Pe_O2_ value, which is equal to 0.46 × 10^−8^ cm^2^/s, and this value is 97.7% lower compared to the Pe_O2_ value of pure LDPE, which is equal to 20.02 × 10^−8^ cm^2^/s.

In summary, the Pe_O2_ values are decreased for all LDPE/xNZ and LDPE/xTO@NZ films compared to the Pe_O2_ values of the pure LDPE film while the D_wv_ values are increased for all LDPE/xNZ films and decreased for all LDPE/xTO@NZ films as compared to the D_wv_ values of the pure LDPE film. The different behaviors of Pe_O2_ and Dwv values when NZ is dispersed into LDPE matrix could be attributed to the different mechanisms followed by water and oxygen molecules when they diffuse through a polymer matrix beause water vapor molecules are polar while O_2_ gas molecules are not [48,49,50,51,52]. Thus, hydrophilic NZ seems to favor the permeation of polar water vapor molecules through the LDPE matrix. In addition, when NZ is dispersed into LDPE, it has been shown to act as a CO_2_ adsorbent, and it is possible that a similar mechanism reduces the oxygen permeability of LDPE/xNZ films [22,23].

Finally, we must emphasize that, to the best of our knowledge, the water and oxygen barrier properties studied and discussed here for LDPE/xNZ and LDPE/xTO@NZ films are being reported for the first time.

### 3.6. Antioxidant Activity of LDPE/xNZ and LDPE/xTO@NZ Films

**Figure 9:**
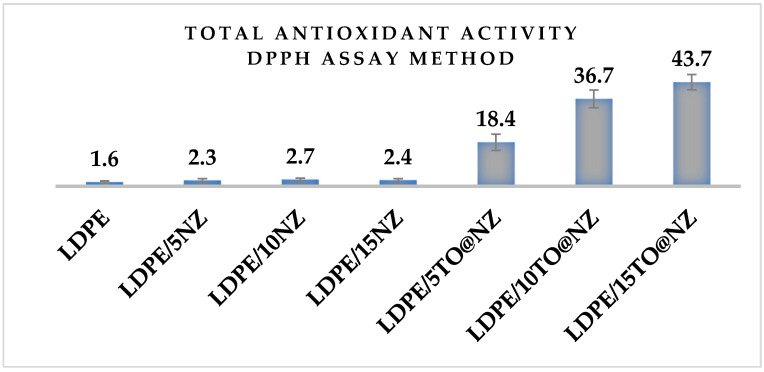
The % total antioxidant activity values after 24 h incubation for all LDPE/xNZ and LDPE/xTO@NZ films, as well as for pure LDPE film.

In the column bar diagram of Figure 9, the determined % antioxidant activity of all LDPE/xNZ and LDPE/xTO@NZ films, as well as pure LDPE film, are presented for comparison.

As can be observed in Figure 8, the antioxidant activity for all LDPE/xNZ films and pure LDPE film is almost negible. On the contrary, all LDPE/xTO@NZ films exhibit significant antioxidant actiity. As expected, the obtained antioxidant activity of LDPE/xTO@NZ films increases as the wt% content of TO@NZ increases. The highest value of antioxidant activity was achieved from LDPE/15TO@NZ film, and it is equal to 43.7%.

### 3.7. Diffusion Coefficient Calculation for Controlled Release of TO

**Figure 10:**
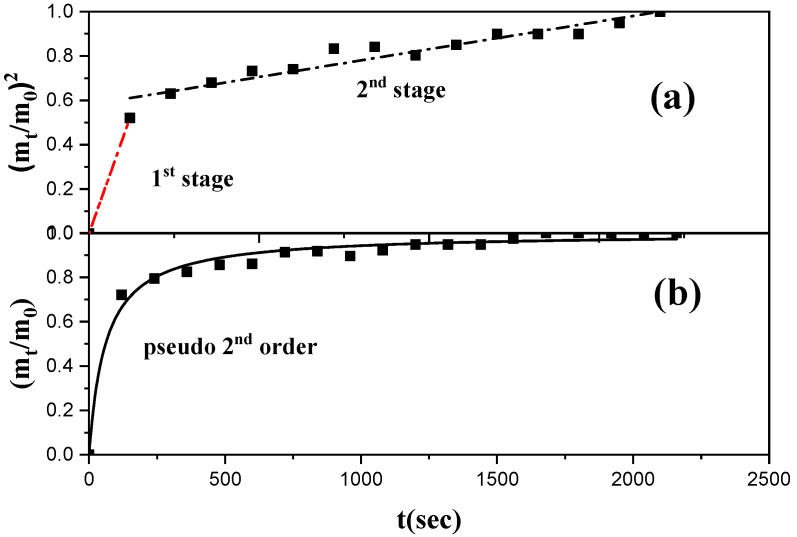
Desorption kinetics of controlled TO release from LDPE/15TO@NZ film: (**a**) linearized mode, Equation (7), and (**b**) pseudo-second-order sorption kinetic model, Equation (8).

The LDPE/15TO@NZ film was chosen to calculate the diffusion coefficient of the release TO (D_TO_). According to the experimental results discussed above, this film has the highest amount of TO, exhibits the highest total antioxidant activity, has the lowest Pe_O2_ value, and its mechanical behavior is similar to the pure LDPE film (see the E, σ_uts_, and %ε values in Table 1). The data from all the thermogravimetric experiments of LDPE/15TO@NZ and LDPE/15NZ films were used to calculate the migration of TO and are shown in Tables S4 and S5 of the Supplementary Materials. From the data of Tables S4 and S5, the total TO released equal to 12.9 ± 2.5 and the values of parameters in Equations (6) and (7) were calculated. Considering that the modified TO@NZ contains 35.5 wt% TO and the film contains 15 wt% TO@NZ, then the nominal content of TO in the tested LDPE/15TO@NZ active films, calculated by multiplication, is 5.325 wt%, which is much higher than the calculated released TO of 2.142 wt%. This fact probably means that the final TO content in the film after the extrusion molding process is lower than 5.325 wt% and that there is probably some amount of TO remaining inside the LDPE matrix which cannot be released. Using the data of Table S6 and according to Equation (7), the (m_0_/m_t_)^2^ vs. time (t) curve was plotted and is presented in Figure 10. From this plot, it is obvious that there are two linear regions, which means that the desorption process of the TO was carried out following the common two-stage desorption path. The first quick stage probably concerns TO molecules which were adsorbed on the external surface of the nanohybrids or which were physiosorbed on the surface of meso-macropores of the nanohybrids. The second, slower stage probably concerns TO molecules which were chemisorbed to the micro-mesopores of the nanohybrids. This hypothesis was also evidenced by the application of the pseudo-second-order sorption kinetic model on the experimental TO controlled release data, which led to the desorption rate constant k_2_ = 0.01647 (s^−1^). The diffusion coefficient of released TO (D_TO_) for the first stage was estimated as 2.09 × 10^−7^ cm^2^·s^−1^ and as 1.21 × 10^−8^ cm^2^·s^−1^ for the second stage. Thus, the second stage of the desorption process seems to be 17–18 times slower than the first stage of the desorption process.

### 3.8. Lipid Oxidation

#### 3.8.1. TBARS

The TBARS values for pork fillets packaged in the control, NZ, and TO@NZ films are shown in Table 4. As can be clearly seen, the samples packed in control pouches had the highest TBARS values throughout the storage period. The higher the value of TBARS, the higher the degree of lipid oxidation in meat. Lipid oxidation is a limiting factor affecting the quality and freshness of meat [53]. However, the TBARS value did not exceed the value of 1.5 mg MDA/kg during the 12 days of storage, in agreement with a recent study [26]. SPSS ver. 20 software was used (IBM SPSS Statistics, IBM Corp., 1 New Orchard Road Armonk, New York 10504-1722, USA) for statistical analysis of the obtained experimental data. It is worth mentioning that the ANOVA showed significant differences in TBARS values at 6, 8, 10, and 12 days of storage; this storage period is of great interest as it has been reported in the literature that this period time is related to the shelf life and deterioration of pork meat using different packaging treatments [53,54,55]. Active packaging has a beneficial effect on the delay of lipid oxidation. In agreement with the results of the present study, Moudache et al. [56] reported that in pork meat wrapped in films containing olive extract, a delay in lipid oxidation was recorded. In a more recent study, Boeira et al. [57] also reported a significant reduction in the lipid oxidation of rump steak packaged in films containing corn stigma residue extracts.

#### 3.8.2. Heme Iron Content

The changes in the heme iron content of pork fillets are shown in Table 4. These changes are an indicator of the lipid oxidation of meat during storage [26,58]. The initial value (day 0) of heme iron content for the pork fillets studied in the present work was 10.41 ± 0.51 μg/g. Previous studies have reported that the heme iron content in different portions of pork meat (loin, topside, shoulder, scaloppini, and rump) ranged from 1.70 to 8.10 μg/g, in general agreement with the results of the present study [26,58,59]. There were significant differences (*p* < 0.05) between the control (LDPE), LDPE/15NZ, and LDPE/15TO@NZ packaging films throughout the storage period. SPSS ver. 20 software was used (IBM SPSS Statistics, IBM Corp., 1 New Orchard Road Armonk, New York 10504-1722, USA), and the ANOVA showed that significant differences (*p* < 0.05) were recorded for days 2, 6, 8, and 10 of storage. The most pronounced results were obtained for the pork fillets packaged with LDPE/15TO@NZ film, in which a significantly (*p* < 0.05) lower reduction in heme iron content was observed throughout storage, especially during the 10th and 12th days of storage. The obtained results indicate that the prepared films containing TO-NT are more effective against the heme iron oxidation of pork fillets compared to the LDPE or LDPE/15NZ packaging films.

It is also noteworthy that, compared to our recent study [26], the lipid oxidation of pork fillets in the present work appeared to have a lower trend during storage time, especially for the LDPE/15TO@NZ packaging films.

#### 3.8.3. Correlation of TBARS and Heme Iron

The TBARS and heme iron content values of pork fillets were subjected to bivariate Pearson’s correlation analysis throughout storage. The results showed significant and positive correlations between the two methods throughout storage. Corresponding correlations are given in the Supplementary Material (see Tables S7–S9) with respect to packaging treatment and storage time. 

### 3.9. Overall Discussion

As shown by the XRD and DSC measurements, the crystallinity of the pure LDPE decreases slightly as a result of the mixing with NZ and TO. However, the fine dispersion of the NZ and especially of the nanohybrid TO@NZ causes an enhanced compatibility of such materials with the polymeric matrix, which is also supported by the FTIR results.

The DSC measurements show that the final product exhibited stable thermal properties, i.e., T_peak_ and ΔH, compared to the initial LDPE matrix. This indicates that the influence of the addition of nanohybrid materials on the behaviour of the LDPE upon heat treatment is negligible and the mixing of such compounds does not induce significant structural modifications. The most stable composite is the LDPE/10TO@NZ film, as shown by the relevant ΔH values. The initial strength of the pure LDPE film was not affected by the incorporation of NZ and TO@NZ nanomaterials, as indicated by the σ_uts_ values, and a higher elasticity was achieved in the case of the LDPE/xNZ and LDPE/xTO@NZ films. Such results were supported by the Young modulus E and the % elongation at break values. The most elastic materials were those with TO@NZ.

It is obvious from the D_WV_ values that in the case of LDPE/xNZ materials, the water vapor barrier decreases slightly compared with the relevant values of pure LDPE; in the case of LDPE/xTO@NZ films, this barrier increases slightly. The oxygen barrier increases drastically in all LDPE/xNZ or LDPE/xTO@NZ cases, as shown by the Pe_O2_ values. The film that exhibited the highest oxygen barrier was the LDPE/15TO@NZ film.

The incorporation of the NZ material into the polymeric matrix slightly increases the film’s antioxidant and antimicrobial activity, while the addition of TO into the mixture increases such properties significantly. This result is supported by both the antioxidant and antimicrobial measurements. The most active material was the LDPE/15TO@NZ film, which exhibited an antioxidant activity of 43.7%. It was proven that the TBARS and heme iron methods provided statistically significantly correlated results. Such correlation seems to be linear, but this conclusion needs further systematic investigation.

Finally, the TO release tests indicated partially physiosorbed and partially chemisorbed TO molecules on the external and internal (porous) surfaces of the NZ, which led to a two-stage desorption process. This result, combined with the improved antioxidant and antimicrobial results, suggests a better preservation and an extension to the shelf life of the pork fillets.

## 4. Conclusions

According to all of the characterization and validation methods used, the incorporation of natural zeolite and the natural thyme oil extract with LDPE, a commonly used food packaging material, results in a promising active packaging film.

The results indicate that an extension to shelf-life and food preservation is possible using natural biodegradable materials instead of chemical such as E-number components.

Future work could be the comparison of our developed films with similar systems and the scale-up of the production process for the best food packaging system.

## Figures and Tables

**Table 1:** Calculated values of enthalpies (ΔH), temperatures (T_start_, T_peak,_ T_end_), and degree of crystallinity (%Xc) for the melting 1, melting 2, and crystallization processes of all LDPE/xNZ and LDPE/15TO@NZ films, as well as pure LDPE film for comparison.

		LDPE	LDPE/ 5NZ	LDPE/ 10NZ	LDPE/ 15NZ	LDPE/ 5TO@NZ	LDPE/ 10TO@NZ	LDPE/ 15TO@NZ
Melting 1	ΔH (J/g)	174.4	143.6	134.2	125.6	129.0	160.9	118.8
	ΔH_LDPE_ (J/g)	174.4	151.2	149.1	147.8	135.8	178.8	139.8
	T_start_ (°C)	103.4	105.0	104.2	95.7	104.7	105.6	105.7
	T_peak_ (°C)	115.0	115.0	114.0	114.0	114.0	114.0	115.0
	T_end_ (°C)	119.0	120.8	121.1	122.0	121.1	119.8	120.7
	%Xc	59.5	51.6	50.9	50.4	46.3	61.0	47.7
Crystallization	ΔH (J/g)	−168.2	−139.2	−128.3	−125.3	−130.9	−151.6	−117.7
	ΔH_LDPE_ (J/g)	−168.2	−146.5	−142.6	−147.4	−137.8	−168.4	−138.5
	T_start_ (°C)	100.0	101.3	99.0	99.4	99.4	99.0	98.8
	T_peak_ (°C)	93.0	93.0	92.0	91.5	92.0	93.0	92.0
	T_end_ (°C)	79.4	77.0	74.8	75.0	74.4	78.7	77.0
	%Xc	57.4	50.0	48.7	50.3	47.0	57.5	47.3
Melting 2	ΔH (J/g)	162.4	118.5	125.5	122.5	134.4	147.6	113.6
	ΔH_LDPE_ (J/g)	162.4	124.7	139.4	144.1	141.5	164.0	133.6
	T_start_ (°C)	98.4	97.7	97.9	95.7	97.8	100.0	96.7
	T_peak_ (°C)	110.0	111.0	112.0	111.0	112.0	111.0	112.0
	T_end_ (°C)	117.7	118.5	119.2	119.6	118.8	118.5	118.8
	%Xc	55.4	42.6	47.6	49.2	48.3	56.0	45.6

**Table 2:** Modulus of elasticity €, ultimate strength (σ_uts_), and elongation at break %ε values for pure LDPE film and for all LDPE/xNZ and LDPE/xTO@NZ films.

Sample Code	E	σ_uts_	ε%
LDPE	183.3 ± 18.8	12.6 ± 0.5	29.3 ± 11.0
LDPE/5NZ	257.0 ± 12.7	13.3 ± 0.4	32.0 ± 12.7
LDPE/10NZ	264.0 ± 11.3	11.3 ± 1.2	12.9 ± 3.5
LDPE/15NZ	242.0 ± 12.9	9.8 ± 0.9	9.3 ± 1.9
LDPE/5TO@NZ	281.8 ± 12.3	13.7 ± 1.4	90.0 ± 11.7
LDPE/10TO@NZ	297.3 ± 14.2	12.2 ± 0.8	89.1 ± 13.2
LDPE/15TO@NZ	281.3 ± 13.8	12.0 ± 1.0	37.4 ± 12.1

**Table 3:** Water vapor transmission rate (WVTR), water vapor diffusion coefficient (D_wv_), oxygen transmission rate (OTR), and oxygen permeability (Pe_O2_) values for all LDPE/xNZ and LDPE/xTO@NZ films, as well for pure LDPE films.

Sample Code	Film Thickness (mm)	Water Vapor Transmission Rate WVTR (10^−7^ gr/(cm^2^·s)	Diffusion Coefficient D_wv_ (10^−4^ cm^2^/s)	Oxygen Transmission Rate OTR (mL/(m^2^·day))	Permeability Coefficient Pe_O2_ (10^−8^ cm^2^/s)
LDPE	0.270 ± 0.010	5.09 ± 0.26	3.06 ± 0.23	6407 ± 320	20.02 ± 8.41
LDPE/5NZ	0.266 ± 0.004	7.27 ± 0.23	4.39 ± 0.81	848 ± 42	2.61 ± 0.13
LDPE/10NZ	0.378 ± 0.010	4.21 ± 0.50	3.66 ± 0.49	303 ± 15	1.32 ± 0.62
LDPE/15NZ	0.410 ± 0.015	4.31 ± 0.33	4.05 ± 0.21	1293 ± 65	6.13 ± 0.31
LDPE/5TO@NZ	0.130 ± 0.015	4.02 ± 0.18	1.14 ± 0.14	1345 ± 67	2.02 ± 0.11
LDPE/10TO@NZ	0.311 ± 0.005	3.86 ± 0.14	2.69 ± 0.91	335 ± 17	1.21 ± 0.06
LDPE/15TO@NZ	0.329 ± 0.010	4.02 ± 0.64	2.94 ± 0.52	122 ± 6	0.46 ± 0.03

**Table 4:** TBARS and heme iron content of pork fillets in different packaging systems with respect to storage time. Differrent letters in its column indicate statistically significant differences at the *p* < 0.05 confidence level, as indicated by Post-hoc multiple comparison tests.

TBARS	Day 0	Day 2	Day 4	Day 6	Day 8	Day 10	Day 12
	AVG ± SD						
	(mg/kg)						
Control (LDPE)	0.17 ^a^ ± 0.01	0.26 ^b^ ± 0.04	0.46 ^c^ ± 0.01	0.66 ^d^ ± 0.03	0.91 ^f^ ± 0.03	1.21 ^i^ ± 0.04	1.31 ^k^ ± 0.02
LDPE/15NZ	-	0.24 ^b^ ± 0.02	0.44 ^c^ ± 0.02	0.63 ^d^ ± 0.02	0.85 ^g^ ± 0.02	1.15 ^i^ ± 0.02	1.30 ^k^ ± 0.03
LDPE/15TO@NZ	-	0.20 ^b^ ± 0.02	0.42 ^c^ ± 0.02	0.54 ^e^ ± 0.02	0.79 ^h^ ± 0.02	1.07 ^j^ ± 0.02	1.20 ^l^ ± 0.03
	**ANOVA**						
F	-	3.680	4.356	21.137	21.446	19.918	11.676
*p*	-	0.091	0.068	0.002	0.002	0.002	0.009
**Fe**	**Day 0**	**Day 2**	**Day 4**	**Day 6**	**Day 8**	**Day 10**	**Day 12**
	**AVG ± SD**						
	**(μg/g)**						
Control (LDPE)	10.41 ^a^ ± 0.51	8.76 ^b^ ± 0.30	7.44 ^d^ ± 0.24	6.28 ^e^ ± 0.23	4.98 ^h^ ± 0.26	3.32 ^j^ ± 0.21	1.66 ^l^ ± 0.21
LDPE/15NZ	-	9.22 ^b^ ± 0.23	7.522 ^d^ ± 0.21	6.74 ^f^ ± 0.09	5.38 ^h^ ± 0.07	3.48 ^j^ ± 0.18	1.86 ^l^ ± 0.12
LDPE/15TO@NZ	-	9.62 ^c^ ± 0.09	7.92 ^d^ ± 0.16	7.10 ^g^ ± 0.09	5.64 ^i^ ± 0.16	3.90 ^k^ ± 0.12	2.10 ^l^ ± 0.24
	**ANOVA**						
F	-	11.112	4.69	22.228	10.110	8.855	3.753
*p*	-	0.010	0.060	0.002	0.012	0.016	0.088

## Data Availability

Data is contained within the article and supplementary material.

## Supplementary Materials

The following supporting information can be downloaded at: https://www.mdpi.com/article/10.3390/antiox12020523/s1, Figure S1: DSC plots of (a) pure LDPE, (b) LDPE/5NZ, (c) LDPE/5TO@NZ, (d) LDPE/10NZ, (e) LDPE/10TO@NZ, (f) LDPE/15NZ and (g) LDPE/15TO@NZ; Table S1: GC-MS analysis of thyme oil as received; Table S2: GC-MS analysis of the collected after the distillation process thyme oil rich in Limonene and p- Cymene fraction; Table S3: GC-MS results of the remaining after the distillation process thyme oil rich in thymol (TO) fraction; Table S4: Mass changes of LDPE/15NZ films as a function of time for temperature variation from 0 to 85 °C to calculate the water vapor content of the control film; Table S5: Mass changes of LDPE/15TO@NZ films as a function of time for temperature variation from 0 to 90 °C in order to calculate the TO content released from the film; Table S6: Calculated values of: slope of equation (5), diffusion coefficient of TO released in mm^2^/s and m^2^s, total film mass loss or total mass of TO released and % Film total weight loss or % total TO content released; Table S7: Pure LDPE control sample; Table S8: LDPE/15NZ sample; Table S9: LDPE/15TO@NZ sample
