# Supplementary material for: Development and Evaluation of a Novel-Thymol@Natural-Zeolite/Low-Density-Polyethylene Active Packaging Film: Applications for Pork Fillets Preservation
Source: Antioxidants (Basel). 2023 Feb 19;12(2):523. doi: 10.3390/antiox12020523 (PMC9952793; doi:10.3390/antiox12020523)
Supplement: Supplementary file 1 [file antioxidants-12-00523-s001.zip › antioxidants-2230436-supplementary.pdf]

Supplementary material

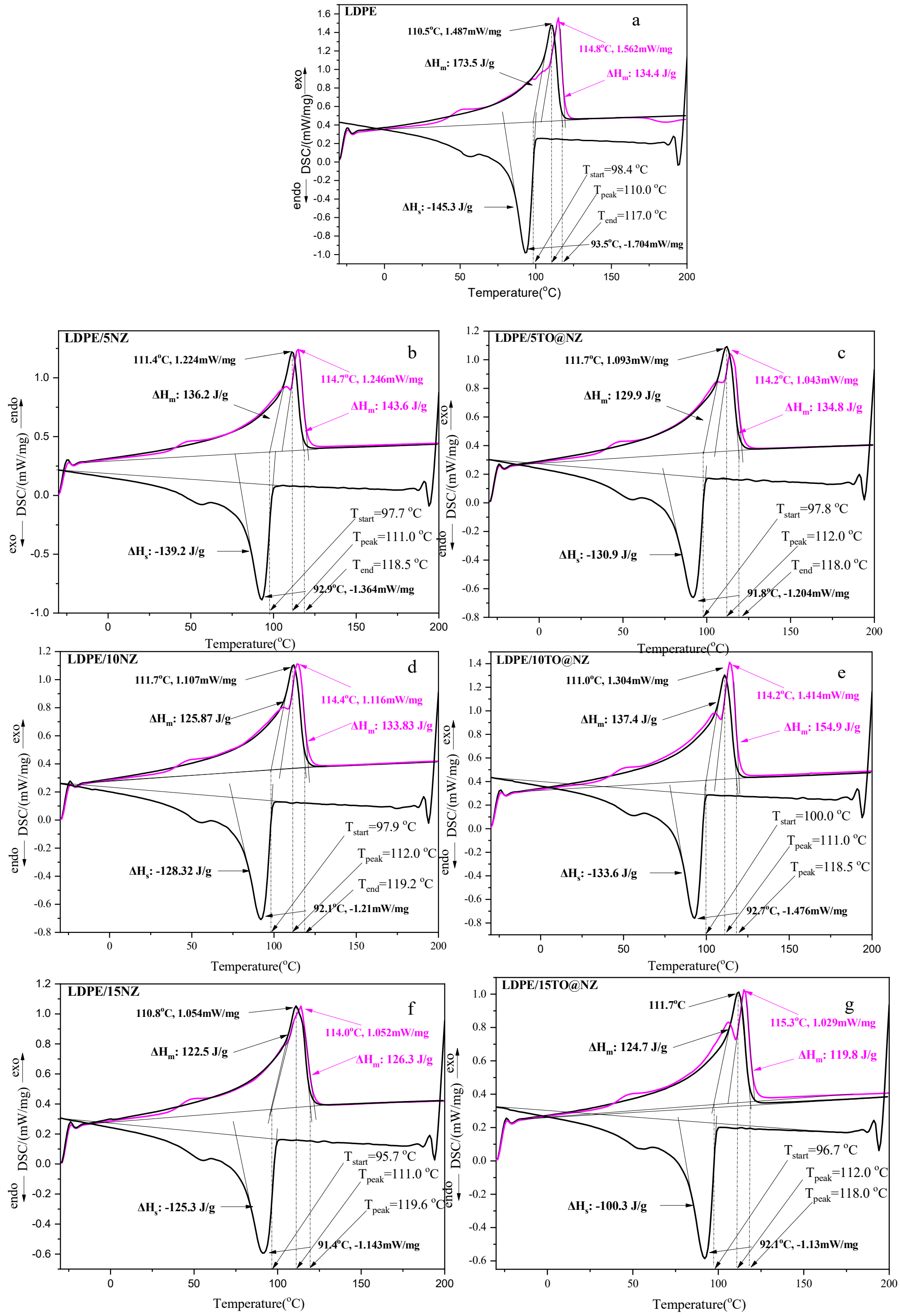

Figure S1. DSC plots of (a) pure LDPE, (b) LDPE/5NZ, (c) LDPE/5TO@NZ, (d) LDPE/10NZ, (e) LDPE/10TO@NZ, (f) LDPE/15NZ and (g) LDPE/15TO@NZ

Table S1. GC-MS analysis of thyme oil as received

Peak	Label	Area Sum %
1	Cpd 1: .ALPHA.-PINENE, (-)-; C10 H16; 6.658	0.81
2	Cpd 2: Camphene; C10 H16; 7.148	0.25
3	Cpd 3: Sabinene; C10 H16; 8.171	0.11
4	Cpd 4: .beta.-Myrcene; C10 H16; 8.864	0.56
5	Cpd 5: Cyclohexene, 1-methyl-4-(1-methylethenyl)-, (R)-; C10 H16; 9.298	0.09
6	Cpd 6: .ALPHA. TERPINENE; C10 H16; 9.802	0.72
7	Cpd 7: Cyclohexane, 1-methyl-3-(1-methylethenyl)-, cis-; C10 H18; 9.998	0.12
**8**	**Cpd 8: Benzene, 1-methyl-4-(1-methylethyl)-; C10 H14; 10.145 ή p-cymene**	**12.28**
**9**	**Cpd 9: D-Limonene; C10 H16; 10.341**	**16.54**
10	Cpd 10: .gamma.-Terpinene; C10 H16; 11.616	0.34
11	Cpd 11: .alpha.-terpinolene; C10 H16; 12.890	0.23
12	Cpd 12: Linalool; C10 H18 O; 13.497	1.67
13	Cpd 13: Camphor; C10 H16 O; 15.292	0.11
14	Cpd 14: Cyclohexanol, 1-methyl-4-(1-methylethenyl)-; C10 H18 O; 15.390	0.08
15	Cpd 15: Bicyclo[2.2.1]heptan-2-ol, 1,7,7-trimethyl-, exo-; C10 H18 O; 15.845	0.1
16	Cpd 16: endo-Borneol; C10 H18 O; 16.265	0.41
17	Cpd 17: .ALPHA.-TERPINEOL; C10 H18 O; 17.462	4.21
18	Cpd 18: Cyclohexanol, 1-methyl-4-(1-methylethylidene)-; C10 H18 O; 17.778	0.52
19	Cpd 19: Fenchyl acetate; C12 H20 O2; 18.821	0.26
20	Cpd 20: Cyclohexanol, 2-methylene-3-(1-methylethyl)-, acetate, cis-; C12 H20 O2; 20.228	0.12
21	Cpd 21: Bicyclo[2.2.1]heptan-2-ol, 1,7,7-trimethyl-, acetate, (1S-endo)-; C12 H20 O2; 21.831	2.89
**22**	**Cpd 22: Phenol, 5-methyl-2-(1-methylethyl)-; C10 H14 O; 22.273 OR THYMOL**	**32.54**
**23**	**Cpd 23: Phenol, 5-methyl-2-(1-methylethyl)-; C10 H14 O; 22.651 OR THYMOL**	**24.16**
24	Cpd 24: Tetradecane; C14 H30; 26.754	0.11
25	Cpd 25: TRANS(.BETA.)-CARYOPHYLLENE; C15 H24; 27.251	0.36
26	Cpd 26: Hexadecane; C16 H34; 34.638	0.17
27	Cpd 27: Octadecane; C18 H38; 41.781	0.07
28	Cpd 28: Hexadecanoic acid, 2-hydroxy-1-(hydroxymethyl)ethyl ester; C19 H38 O4; 60.105	0.19

Table S2. GC-MS analysis of the collected after the distillation process thyme oil rich in Limonene and p- Cymene fraction.

Label	Area Sum %
Cpd 1: l-Phellandrene; C10 H16; 6.462	0.15
Cpd 2: .ALPHA.-PINENE, (-)-; C10 H16; 6.658	3.7
Cpd 3: Camphene; C10 H16; 7.141	1.52
Cpd 4: 2-.BETA.-PINENE; C10 H16; 8.171	0.35
Cpd 5: .beta.-Myrcene; C10 H16; 8.864	1.68
Cpd 6: Cyclohexane, 1-methylene-4-(1-methylethenyl)-; C10 H16; 9.263	0.18
Cpd 7: 3-Carene; C10 H16; 9.522	0.1
Cpd 8: .ALPHA. TERPINENE; C10 H16; 9.809	0.33
Cpd 9: Benzene, 1-methyl-4-(1-methylethyl)-; C10 H14; 10.194-p cymene	**31.98**
Cpd 10: D-Limonene; C10 H16; 10.397	**31.83**
Cpd 11: .gamma.-Terpinene; C10 H16; 11.623	0.07
Cpd 12: 12.918	0.15
Cpd 13: Linalool; C10 H18 O; 13.499	2.09
Cpd 14: D-Fenchyl alcohol; C10 H18 O; 13.940	0.08
Cpd 15: 3-Cyclohexen-1-ol, 1-methyl-4-(1-methylethyl)-; C10 H18 O; 14.935	0.22
Cpd 16: Cyclohexanol, 1-methyl-4-(1-methylethenyl)-; C10 H18 O; 15.390	0.12
Cpd 17: 15.845	0.19
Cpd 18: endo-Borneol; C10 H18 O; 16.258	0.3
Cpd 19: 3-Cyclohexen-1-ol, 4-methyl-1-(1-methylethyl)-; C10 H18 O; 16.825	0.06
Cpd 20: .ALPHA.-TERPINEOL; C10 H18 O; 17.453	1.77
Cpd 21: Cyclohexanol, 1-methyl-4-(1-methylethylidene)-; C10 H18 O; 17.777	0.23
Cpd 22: Fenchyl acetate; C12 H20 O2; 18.814	0.14
Cpd 23: Isobornyl acetate; C12 H20 O2; 21.762	0.63
**Cpd 24: Phenol, 5-methyl-2-(1-methylethyl)-; C10 H14 O; 22.210**	**13.31**
**Cpd 25: Phenol, 5-methyl-2-(1-methylethyl)-; C10 H14 O; 22.574**	**8.32**
Cpd 26: Tetradecane; C14 H30; 26.754	0.09
Cpd 27: Hexadecane; C16 H34; 34.638	0.15
Cpd 28: Hexadecanoic acid, 2-hydroxy-1-(hydroxymethyl)ethyl ester; C19 H38 O4; 60.105	0.23

Table S3. GC-MS results of the remaining after the distillation process thyme oil rich in thymol (TO) fraction

Peak	Label	Area Sum %
1	Cpd 1: Camphene; C10 H16; 7.142	0.18
2	Cpd 2: Benzene, 1-methyl-4-(1-methylethyl)-; C10 H14; 10.131	0.99
3	Cpd 3: D-Limonene; C10 H16; 10.292	0.97
4	Cpd 4: Linalool; C10 H18 O; 13.492	0.69
5	Cpd 5: 3-Cyclohexen-1-ol, 1-methyl-4-(1-methylethyl)-; C10 H18 O; 14.942	0.21
6	Cpd 6: Camphor; C10 H16 O; 15.278	0.15
7	Cpd 7: Cyclohexanol, 1-methyl-4-(1-methylethenyl)-; C10 H18 O; 15.383	0.17
**8**	Cpd 8: 15.838	0.19
**9**	Cpd 9: Bicyclo[2.2.1]heptan-2-ol, 1,7,7-trimethyl-, exo-; C10 H18 O; 16.258	0.54
10	Cpd 10: 3-Cyclohexen-1-ol, 4-methyl-1-(1-methylethyl)-; C10 H18 O; 16.825	0.11
11	Cpd 11: .ALPHA.-TERPINEOL; C10 H18 O; 17.463	1.65
12	Cpd 12: Cyclohexanol, 1-methyl-4-(1-methylethylidene)-; C10 H18 O; 17.778	0.54
13	Cpd 13: 18.611	0.09
14	Cpd 14: Fenchyl acetate; C12 H20 O2; 18.821	0.54
15	Cpd 15: Ascaridole; C10 H16 O2; 20.249	0.19
16	Cpd 16: Isobornyl acetate; C12 H20 O2; 21.748	0.36
17	Cpd 17: Bicyclo[2.2.1]heptan-2-ol, 1,7,7-trimethyl-, acetate, (1S-endo)-; C12 H20 O2; 21.867	3.38
18	**Cpd 18: Phenol, 5-methyl-2-(1-methylethyl)-; C10 H14 O; 22.294**	**46.61**
19	**Cpd 19: Phenol, 5-methyl-2-(1-methylethyl)-; C10 H14 O; 22.679**	**40.09**
20	Cpd 20: 25.592	0.18
21	Cpd 21: Tetradecane; C14 H30; 26.747	0.15
**22**	Cpd 22: TRANS(.BETA.)-CARYOPHYLLENE; C15 H24; 27.251	0.33
**23**	Cpd 23: 29.282	0.18
24	Cpd 24: CARYOPHYLLENE OXIDE; C15 H24 O; 33.672	0.1
25	Cpd 25: Hexadecane; C16 H34; 34.639	0.21
26	Cpd 26: 48.132	0.37
27	Cpd 27: 48.426	0.39
28	Cpd 28: 60.105	0.43

Table S4. Mass changes of LDPE/15NZ films as a function of time for temperature variation from 0 to 85 °C to calculate the water vapor content of the control film.

	LDPE/15NZ_1		LDPE/15NZ_2		LDPE/15NZ_3		LDPE/15NZ
	average film thickness l 0.061 mm		average film thickness l 0.119 mm		average film thickness l 0.0781 mm		-
Time (s)	m_t (mg)	Δm (m_t-m_0)	m_t (mg)	Δm (m_t-m_0)	m_t (mg)	Δm (m_t-m_0)	average Δm / water vapor
0	520	0	690	0	605	0	0
120	517	3	688	2	603	3	3
240	516	4	688	2	602	3	3
360	516	4	687	3	602	4	4
480	516	4	686	4	601	4	4
600	516	4	685	5	601	5	5
720	516	4	685	5	601	5	5
840	516	4	684	6	600	5	5
960	516	4	683	7	600	6	6
1080	516	4	683	7	600	6	6
1200	516	4	683	7	600	6	6
1320	516	4	683	7	600	6	6
1440	516	4	683	7	600	6	6
1560	516	4	683	7	600	6	6
1680	516	4	683	7	600	6	6
1800	516	4	683	7	600	6	6
1920	516	4	683	7	600	6	6
2040	516	4	683	7	600	6	6
2160	516	4	683	7	600	6	6

Table S5. Mass changes of LDPE/15TO@NZ films as a function of time for temperature variation from 0 to 90 °C in order to calculate the TO content released from the film.

	LDPE/15TO@NZ_1				LDPE/15TO@NZ_2				LDPE/15TO@NZ_3			
	average film thickness l 0.075 mm				average film thickness l 0.074 mm				Average film thickness l 0.086 mm			
**Time (s)**	**m_t (mg)**	**m_t + water vapor**	**m_t / m_∞**	**(m_t / m_∞)^2**	**m_t (mg)**	**m_t + water vapor**	**m_t / m_∞**	**(m_t / m_∞)^2**	**m_t (mg)**	**m_t + water vapor**	**m_t / m_∞**	**(m_t / m_∞)^2**
0	591	591	1.037	1.075	545	545	1.036	1.074	665	665	1.037	1.076
120	580	583	1.022	1.044	534	537	1.020	1.040	654	657	1.024	1.049
240	579	582	1.021	1.043	533	536	1.019	1.038	652	655	1.022	1.044
360	579	583	1.022	1.044	532	536	1.018	1.036	651	655	1.021	1.043
480	578	582	1.021	1.043	532	536	1.019	1.038	650	654	1.020	1.041
600	578	583	1.022	1.044	532	537	1.020	1.040	649	654	1.020	1.039
720	578	583	1.022	1.044	531	536	1.018	1.036	648	653	1.018	1.036
840	577	582	1.021	1.043	531	536	1.019	1.038	648	653	1.019	1.038
960	577	583	1.022	1.044	531	537	1.020	1.040	648	654	1.020	1.039
1080	577	583	1.022	1.044	531	537	1.020	1.040	647	653	1.018	1.036
1200	576	582	1.020	1.041	531	537	1.020	1.040	647	653	1.018	1.036
1320	576	582	1.020	1.041	531	537	1.020	1.040	647	653	1.018	1.036
1440	576	582	1.020	1.041	531	537	1.020	1.040	647	653	1.018	1.036
1560	576	582	1.020	1.041	531	537	1.020	1.040	646	652	1.016	1.033
1680	575	581	1.018	1.037	531	537	1.020	1.040	646	652	1.016	1.033
1800	575	581	1.018	1.037	531	537	1.020	1.040	646	652	1.016	1.033
1920	575	581	1.018	1.037	531	537	1.020	1.040	646	652	1.016	1.033
2040	575	581	1.018	1.037	531	537	1.020	1.040	646	652	1.016	1.033
2160	575	581	1.018	1.037	531	537	1.020	1.040	646	652	1.016	1.033

Table S6. Calculated values of: slope of equation (5), diffusion coefficient of TO released in mm2/s and m2s, total film mass loss or total mass of TO released and % Film total weight loss or % total TO content released.

sample	Slope ($4\frac{D.t}{\pi.l^2}$)	D (mm^2/s)	D(cm^2/s)	Total film mass loss m_0 - m_∞ or total mass of TO released (mg)	% Film total weight loss (m_0 - m_∞)/m_0*100 or % total TO content released
LDPE/15TO@NZ_1	10^{-4}	4.42E-07	$4.42.10^{-10}$	10.0	1.69
LDPE/15TO@NZ_2	10^{-4}	4.29866E-07	$4.30.10^{-10}$	8.0	1.47
LDPE/15TO@NZ_3	10^{-4}	5.80586E-07	$5.81.10^{-10}$	13.0	1.95
average	-	-	$4.84.10^{-13}\pm0.84.10^{-13}$	10.3±2.5	1.70±0.24

Pearson's Correlations

Table S7. pure LDPE control sample

Confidence Intervals

	Pearson Correlation	Sig. (2-tailed)	95% Confidence Intervals (2-tailed)[a]	
			Lower	Upper
DAY0 - DAY2	,998	,000	,976	1,000
DAY0 - DAY4	,998	,000	,977	1,000
DAY0 - DAY6	,995	,000	,939	,999
DAY0 - DAY8	,992	,000	,905	,999
DAY0 - DAY10	,998	,000	,971	1,000
DAY0 - DAY12	,818	,047	-,062	,976
DAY2 - DAY4	1,000	,000	1,000	1,000
DAY2 - DAY6	,998	,000	,975	1,000
DAY2 - DAY8	,997	,000	,963	1,000
DAY2 - DAY10	,995	,000	,942	,999
DAY2 - DAY12	,839	,037	,004	,979
DAY4 - DAY6	,998	,000	,975	1,000
DAY4 - DAY8	,997	,000	,963	1,000
DAY4 - DAY10	,995	,000	,943	,999
DAY4 - DAY12	,838	,037	,000	,979
DAY6 - DAY8	1,000	,000	,994	1,000
DAY6 - DAY10	,987	,000	,860	,998
DAY6 - DAY12	,815	,048	-,072	,975
DAY8 - DAY10	,984	,000	,826	,998
DAY8 - DAY12	,823	,044	-,048	,976
DAY10 - DAY12	,843	,035	,015	,979

a. Estimation is based on Fisher's r-to-z transformation with bias adjustment.

Table S8. LDPE/15NZ sample

Confidence Intervals

	Pearson Correlation	Sig. (2-tailed)	95% Confidence Intervals (2-tailed)[a]	
			Lower	Upper
DAY0 - DAY2	,999	,000	,986	1,000
DAY0 - DAY4	,998	,000	,980	1,000
DAY0 - DAY6	,997	,000	,969	1,000
DAY0 - DAY8	,999	,000	,983	1,000
DAY0 - DAY10	,998	,000	,980	1,000
DAY0 - DAY12	,979	,001	,783	,997
DAY2 - DAY4	1,000	,000	,999	1,000
DAY2 - DAY6	,999	,000	,993	1,000
DAY2 - DAY8	,999	,000	,989	1,000
DAY2 - DAY10	,995	,000	,946	,999
DAY2 - DAY12	,973	,001	,726	,997
DAY4 - DAY6	,999	,000	,993	1,000
DAY4 - DAY8	,999	,000	,985	1,000
DAY4 - DAY10	,994	,000	,933	,999
DAY4 - DAY12	,972	,001	,717	,996
DAY6 - DAY8	,999	,000	,993	1,000
DAY6 - DAY10	,994	,000	,936	,999
DAY6 - DAY12	,965	,002	,659	,996
DAY8 - DAY10	,997	,000	,968	1,000
DAY8 - DAY12	,968	,002	,683	,996
DAY10 - DAY12	,975	,001	,747	,997

a. Estimation is based on Fisher's r-to-z transformation with bias adjustment.

Table S9. LDPE/15TO@NZ sample

Confidence Intervals				
	Pearson Correlation	Sig. (2-tailed)	95% Confidence Intervals (2-tailed)[a]	
			Lower	Upper
DAY0 - DAY2	,999	,000	,983	1,000
DAY0 - DAY4	,999	,000	,986	1,000
DAY0 - DAY6	,999	,000	,983	1,000
DAY0 - DAY8	,997	,000	,969	1,000
DAY0 - DAY10	,998	,000	,972	1,000
DAY0 - DAY12	,940	,005	,474	,992
DAY2 - DAY4	,999	,000	,994	1,000
DAY2 - DAY6	1,000	,000	1,000	1,000
DAY2 - DAY8	1,000	,000	,995	1,000
DAY2 - DAY10	,999	,000	,991	1,000
DAY2 - DAY12	,956	,003	,583	,994
DAY4 - DAY6	,999	,000	,992	1,000
DAY4 - DAY8	,998	,000	,978	1,000
DAY4 - DAY10	,998	,000	,972	1,000
DAY4 - DAY12	,948	,004	,524	,993
DAY6 - DAY8	1,000	,000	,996	1,000
DAY6 - DAY10	,999	,000	,993	1,000
DAY6 - DAY12	,956	,003	,587	,994
DAY8 - DAY10	1,000	,000	,997	1,000
DAY8 - DAY12	,962	,002	,635	,995
DAY10 - DAY12	,961	,002	,624	,995

a. Estimation is based on Fisher's r-to-z transformation with bias adjustment.
